# A Significant Step Forward in Understanding Human Milk: the Mothers, Infants, and Lactation Quality Study^☆^

**DOI:** 10.1016/j.advnut.2025.100509

**Published:** 2025-10-28

**Authors:** Andrew A Bremer

**Affiliations:** Office of Nutrition Research, National Institutes of Health, Bethesda, MD, United States

Breastfeeding is universally recognized as the optimal method of infant feeding, providing essential nutrients and antibodies that support healthy growth and development [1]. The WHO recommends exclusive breastfeeding for the first 6 mo of life and continued breastfeeding for ≤2 y or beyond [2]. However, despite these recommendations, there remains a significant gap in reliable data on the nutrient composition of human milk. This gap presents a major challenge in accurately establishing nutrient intake recommendations for infants and lactating women.

The Mothers, Infants, and Lactation Quality (MILQ) study—the results of which are presented in this Supplement of *Advances in Nutrition*—addresses this gap by establishing reference values (RVs) for nutrient concentrations in human milk through the first 8.5 mo of lactation [[3], [4], [5], [6], [7], [8], [9]]. This multicenter study—led by experts in the field of human milk research and conducted across diverse geographical sites in Bangladesh, Brazil, Denmark, and The Gambia—represents the most comprehensive effort to date in understanding the nutrient profile of human milk from well-nourished mothers. The study team should be congratulated for their efforts; this study will not only be the benchmark for future studies in the human milk composition space but will also inform present nutrient intake recommendations for infants and lactating women worldwide.

The primary objective of the MILQ study was to provide global RVs for macro- and micronutrients in human milk. Importantly, the investigators also measured milk volume to quantify nutrient transfer to the infant—crucial data for accurately assessing an infant’s nutrient intake and adjusting the recommended nutrient intake for the lactating woman. The MILQ study enrolled a total of 1242 mother–infant dyads across the 4 sites—an impressive accomplishment indeed. The study began in 2017 and concluded in 2022; sample collections occurred at specific postpartum intervals: colostrum (0–3 d), and milk at 1–3.49, 3.5–5.99, and 6–8.5 mo postpartum. An add-on study, early MILQ (E-MILQ), was designed to capture milk composition during the first month of lactation, a period characterized by rapid changes in nutrient concentrations.

The MILQ study sites were chosen based on criteria ensuring well-nourished populations, cultural practices of exclusive and prolonged breastfeeding, minimal maternal nutrient supplementation or dietary fortification, and the presence of experienced investigators in perinatal research. The sample size calculations also ensured accurate estimations of nutrient concentrations and comparability across sites. Maternal and infant inclusion criteria were also stringent. Furthermore, data collection was meticulously standardized across sites, and biological samples—including milk, blood, urine, and stool—were collected alongside comprehensive anthropometric and dietary data at multiple time points. Milk samples were collected using the Medela Symphony electric hospital-grade breast pump (Medela), and advanced analytical methods were employed to measure nutrient concentrations [10].

Specifically, liquid chromatography-tandem MS (LC-MS/MS) was used for quantifying B vitamins and vitamin D [11], whereas near-infrared (NIR) spectrometry measured macronutrients [12]. Inductively coupled plasma-MS (ICP-MS) further allowed simultaneous analysis of multiple minerals [13]. Milk volume was measured using the stable isotope dilution dose-to-mother method, endorsed by the International Atomic Energy Agency [14,15].

Overall, the MILQ study’s strengths lie in its systematic data collection across a comprehensive lactation period, stringent inclusion criteria ensuring well-nourished participants, standardized milk collection protocols, advanced analytical methods, and accurate measurement of milk volume. These strengths collectively contribute to the reliability and applicability of the RVs established by the study. However, the study also faced limitations. Specifically, cultural sensitivity required occasional modifications to data collection methods between sites. In Denmark, for instance, milk volume was measured by test-weighing rather than the deuterium oxide method. Additionally, the COVID-19 pandemic resulted in recruitment delays and increased loss-to-follow-up.

Furthermore, although the study was geographically diverse, the results may not be completely globally representative, as they reflect the nutrient composition of milk from well-nourished mothers in specific regions.

Despite its limitations, the MILQ study is the most comprehensive study of its kind and will have profound implications for global health. Specifically, the MILQ study will provide an evidence base to address the following:

• Updating nutrient intake recommendations: The RVs established by the MILQ study provide a robust foundation for updating nutrient intake recommendations for infants and lactating women. This is crucial for ensuring that dietary guidelines are based on accurate and representative data.
• Understanding nutrient variability: The observed variability in nutrient concentrations in the MILQ study underscores the need for context-specific approaches to nutrition assessment and intervention. Recognizing that human milk composition may vary based on geographical and temporal factors is also essential for developing tailored nutrition programs that meet the specific needs of different populations.
• Informing public health strategies: The data from the MILQ study may be used to inform public health strategies aimed at improving maternal and infant nutrition. For example, the findings may guide interventions to address nutrient deficiencies in populations at risk—potentially through targeted supplementation or fortification programs.
• Advancing human milk research: The MILQ study sets a new standard for research on human milk composition, providing a comprehensive data set for future studies. The integration of data on maternal and infant biomarkers, the microbiome, and metabolomics offers new opportunities for exploring the complex interactions between diet, health, and human milk composition.

The MILQ study represents a landmark effort in understanding the nutrient composition of human milk. Specifically, the data provide a critical resource for updating nutrient intake recommendations for infants and lactating women, evaluating infant nutrition strategies, and assessing interventions to optimize maternal and infant nutritional status. Moving ahead, research may focus on exploring the variability in milk nutrient concentrations—investigating the influence of maternal diet, inflammation, and genetic polymorphisms. Additionally, future studies may aim to quantify the impact of maternal micronutrient supplementation on milk composition, using the RVs established by the MILQ study as benchmarks.

To summarize, the MILQ study offers a comprehensive and reliable data set that addresses longstanding gaps in our understanding of human milk composition. These findings will undoubtedly contribute to improving global recommendations for infant and maternal nutrition, ultimately supporting better health outcomes for mothers and their infants.

## Funding

The author reported no funding received for this study.

## Conflict of interest

The author reports no conflicts of interest. AAB is an Editor for *Advances in Nutrition* and played no role in the Journal’s evaluation of the manuscript.
